# The Effect of Microwaves on Protein Structure: Molecular Dynamics Approach

**DOI:** 10.1021/acs.jcim.3c01937

**Published:** 2024-03-13

**Authors:** Matic Broz, Chris Oostenbrink, Urban Bren

**Affiliations:** † Faculty of Chemistry and Chemical Engineering, University of Maribor, Smetanova ulica 17, Maribor SI-2000, Slovenia; ‡ Institute of Molecular Modeling and Simulation, University of Natural Resources and Life Sciences, Muthgasse 18, Vienna 1190, Austria; § Faculty of Mathematics, Natural Sciences and Information Technologies, University of Primorska, Glagoljaška ulica 8, Koper SI-6000, Slovenia; ∥ Institute of Environmental Protection and Sensors, Beloruska ulica 7, Maribor SI-2000, Slovenia

## Abstract

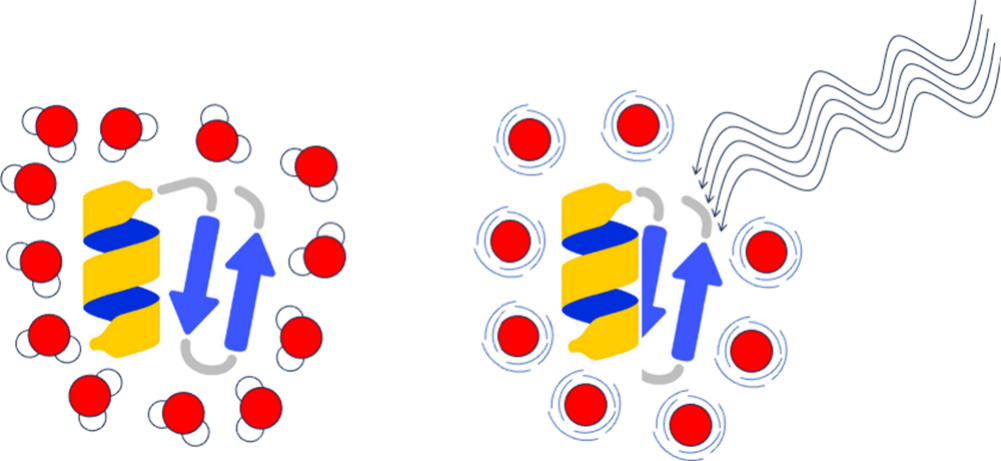

The impact of microwave (MW) irradiation on protein folding, potentially inciting misfolding, was investigated by employing molecular dynamics (MD) simulations. Twenty-nine proteins were subjected to MD simulations under equilibrium (300 K) and MW conditions, where the rotational temperature was elevated to 700 K. The utilized replacement model captures the microwave effects of δ- and γ-relaxation processes (frequency range of ∼300 MHz to ∼20 GHz). The results disclosed that MW heating incited a shift toward more compact protein conformations, as indicated by decreased root-mean-square deviations, root-mean-square fluctuations, head-to-tail distances, and radii of gyration. This compaction was attributed to the intensification of intramolecular electrostatic interactions and hydrogen bonds within the protein caused by MW-destabilized hydrogen bonds between the protein and solvent. The solvent-accessible surface area (SASA), particularly that of polar amino-acid residues, shrank under MW conditions, corresponding to a reduced polarity of the water solvent. However, MW irradiation produced no significant alterations in protein secondary structures; hence, MW heating was observed to primarily affect the protein tertiary structures.

## 1 Introduction

Protein folding, a critical determinant of correct protein functionality within cells, is a subject of immense scientific inquiry due to its profound implications for understanding cellular processes and treating an array of maladies.^1−4^ The delicately maintained tertiary structure of peptides and proteins in solution results from a competition between intramolecular torsional bending and nonbonded interactions, such as hydrogen bonds, salt bridges, and hydrophobic interactions, under the influence of the surrounding solvent.

The potential influence of environmental factors, particularly microwave (MW) radiation, on protein folding has drawn significant attention.^5−10^ MW radiation, a nonionizing electromagnetic radiation prevalent in many modern applications, is suspected of having notable impacts on cellular structures and functions^11,12^ by altering the structural dynamics of biomacromolecular chains such as peptides,^13^ proteins,^8,9,11^ DNA,^14,15^ and RNA,^16^ the aberrant processes generally associated with the onset of neurodegenerative disorders and certain types of cancer.^17−25^

Two potential modalities of the influence of MW on protein folding have been postulated. The first is the induction of equilibrium effects with microwaves raising the temperature of exposed materials. Given that protein folding and stability are temperature-dependent, it is plausible that microwave-induced heating could perturb these processes.^26^ On the other hand, microwave radiation might instigate nonequilibrium effects, potentially disrupting the balance of forces governing protein folding by inducing molecular vibrations or rotations.^27−29^ Auerbach and co-workers indeed provided convincing experimental evidence using quasielastic neutron scattering measurements that in a MW-irradiated system, the rotational temperature may substantially exceed the translational one.^30^

The exploration of the impact of MW on protein folding has yielded intriguing insights. A novel mechanism of microwave catalysis, based on rotationally excited polar reactive species, has been proposed and validated through computer simulations of neutral ester hydrolysis.^31^ This mechanism suggests a reduced activation free energy when the rotational temperature exceeds the translational temperature, indicating a catalytic effect.^31^ Further work has provided an analytical solution of microwave catalysis, aligning with Monte Carlo simulations and experimental observations in polyethylene terephthalate solvolysis.^32^ Nonequilibrium molecular dynamics (MD) simulations have been used to investigate the dynamics of hydrogen bonds in bulk water under MW heating.^33−36^ These studies have shown that an increased rotational temperature modifies the average path of the hydrogen-bond switch and decreases the decay times of water molecule reorientation.

We recently reported novel findings concerning the effect of MW radiation on the conformational preferences of a small helical β-peptide.^13^ We found that while conventional heating leads to a total loss of structure, MW heating precipitates a more subtle shift in the conformational equilibrium. This shift is attributed to the rotationally excited water molecules under MW radiation, which form fewer hydrogen bonds with the peptide. Consequently, the peptide retains more intramolecular interactions, enabling it to maintain stable compact conformations. Moreover, these changes were also observed to trigger the formation of previously unseen misfolded structures not present under conventional heating, indicating the potential of MW radiation to act as a catalyst for peptide and protein misfolding.

In the current MD simulations of 29 proteins, we observed changes in protein structural properties when the rotational temperature of water was increased to 700 K, with the translational temperature held at 300 K. This approach effectively mimicked the δ- and γ-relaxation processes typically observed in the dielectric spectra of protein solutions within the MW frequency range of approximately 300 MHz to 20 GHz.^37^ We observed a strengthened intramolecular hydrogen bond network, leading to more compact protein configurations, as indicated by reduced root-mean-square deviation (RMSD), root-mean-square fluctuation (RMSF), and radius of gyration (RGYR) values. Proteins also displayed a decreased solvent-accessible surface area (SASA), particularly for polar residues, although the secondary structure remained largely unchanged. This can be ascribed to the water solvent exhibiting a less polar and less protic nature under MW conditions.

## 2 Computational Methods

We conducted MD simulations of 29 proteins under two conditions: equilibrium and MW conditions. The 29 protein structures were chosen based on the simulation efficiency from the larger set of 52 proteins described in the PhD thesis of Martina Setz,^38^ who selected X-ray diffraction (crystal) structures based on the following parameters: resolution ≤1.5 Å, no coordinated ions or ligands, no DNA- or RNA-binding, monomer in solution, and less than 250 amino-acid residues. Crystal structures not matching the resolution criterion were also chosen if they had a paired NMR structure. This protein set was used in the work of Diem and Oostenbrink as well to characterize the effects of backbone reparameterization^39^ and of different choices to compute nonbonded interactions.^40^Table S1 in the Supporting Information lists all selected X-ray diffraction and NMR structures.

### 2.1 Preprocessing

To prepare the protein structures for MD simulations, hydrogen atoms were added where necessary using the molecular geometry to position them. If a structure had missing coordinates for heavy atoms, an initial energy minimization of these missing atoms was conducted *in vacuo*: all atoms except the missing ones were position-constrained, and Lennard-Jones interactions were turned off (but the charges were kept), so the atoms could move more freely. For all proteins, an energy minimization was performed by using the steepest descent algorithm with an initial step size of 0.01 nm and a maximum step size of 0.05 nm. A minimum of 100 and a maximum of 1000 minimization steps were made. The energy convergence threshold was 0.001 kJ/mol for minimizations *in vacuo* and 0.01 kJ/mol in solvent. The reaction field method^41^ was used for long-range electrostatic interactions beyond a cutoff radius of 1.4 nm. The reaction field relative permittivity was set to 61. Forces on bonds were explicitly calculated (no bond length constraints were used). If present, crystal waters were energy minimized with 100 *in vacuo* steps using the position-constrained solute. To relax the whole system, the energy was minimized again *in vacuo* without any position restraints for a maximum of 100 steps. If heavy atoms were originally missing, the maximum number of steps was increased to 1000. Then, the system was solvated in a rectangular box with a minimum distance of 0.23 nm between any existing atom and the center of geometry of any added solvent molecule. Proteins were solvated in SPC water using a 1.2 nm distance between the box wall and the protein. Periodic boundary conditions were imposed, and the solvent was energy minimized with position-restrained solute by using a harmonic function with a force constant of 25 MJ/(mol·nm^2^). Subsequently, ions were added by randomly replacing water molecules more than 0.4 nm away from any protein atom. For protein simulations, Na^+^ and Cl^–^ ions were added at a physiological saline concentration of 0.15 mol/L each, calculated from the number of water molecules in the box. If necessary, Na^+^ or Cl^–^ ions were removed to balance the net charge of the protein and to achieve electroneutrality in the simulated system.

### 2.2 Equilibration

Each equilibration step or cycle was 20 ps long and consisted of 10,000 MD simulation steps with a step size of 2 fs. The solvated structures were heated from 50 to 300 K in increments of 50 K. For the structures exposed to MW radiation, an additional equilibration step was included that increased the rotational bath temperature to 700 K. The SPC water model characterizes water as a rigid molecule, meaning the nine degrees of freedom (dof) of a water molecule are reduced to six dof by fixing three (two bonds and one bond angle), which do not contribute to the kinetic energy. The remaining dof can be divided into three translational dofs and three rotational dof. This allows us to heat the rotational and translational degrees of freedom separately, with the rotational temperature increased to 700 K to mimic the effect of MW irradiation. Simultaneously, the force constant of the harmonic position restraint on the solute atoms was reduced by one-tenth in each step, starting from an initial value of 25 MJ/(mol·nm^2^). The center of mass translation of all atoms was removed after 1000 MD simulation steps. After the heating cycles, roto-translational constraints on the solute atoms were initialized in the last cycle.^42^ Finally, the pressure coupling to 1 atm was switched on in the last cycle. In total, 160 ps were used for equilibration to 300 K and 180 ps for the equilibration of the system exposed to MW radiation.

### 2.3 Production Run

The MD simulations were performed at constant temperature and volume at different temperatures using the GROMOS software package^43^ in combination with the GROMOS 54A8 united-atom force field^44^ and the GROMOS-compatible SPC water model.^45^ We employed the leapfrog integration scheme with a 2 fs time step to solve the equations of motion. The SHAKE algorithm^46^ was applied to constrain solute bond lengths with a relative geometric tolerance of 10^–4^, while the SETTLE algorithm^47^ was utilized to constrain solvent bond lengths and angles. The GROMOS software facilitated a separate coupling of translational and internal-rotational dof. Three distinct heat baths were employed for (1) the dof of solute, (2) the rotational, and (3) the translational dofs of the solvent. Since the SPC water model molecules are rigid, they lack vibrational dofs.

The weak-coupling thermostat^48^ maintained the temperatures of all three baths at 300 K for equilibrium simulation conditions. For nonequilibrium simulations of MW heating, only the rotational dof temperature of solvent molecules was raised to 700 K, with the remaining two heat baths held at 300 K. We based the selected rotational temperature of 700 K on previous studies,^13,49^ estimating the MW power required to maintain 1 mol of water at 700 K rotational temperature, which aligns well with a typical power of MW reactors of around 1000 W. A MW reactor projecting 1000 W of microwaves onto a 30 by 40 cm area corresponds to the electric field strength amplitude (*E*_0_) of 2.51 × 10^3^ V/m. To counteract the energy dissipation in nonequilibrium MW simulations of condensed matter, we applied a relatively short relaxation time, τ, of 0.01 ps.

Production run MD simulations were performed for 100 ns, both at equilibrium 300 K conditions and for the systems subjected to MW radiation.

## 3 Results and Discussion

**Figure 1:**
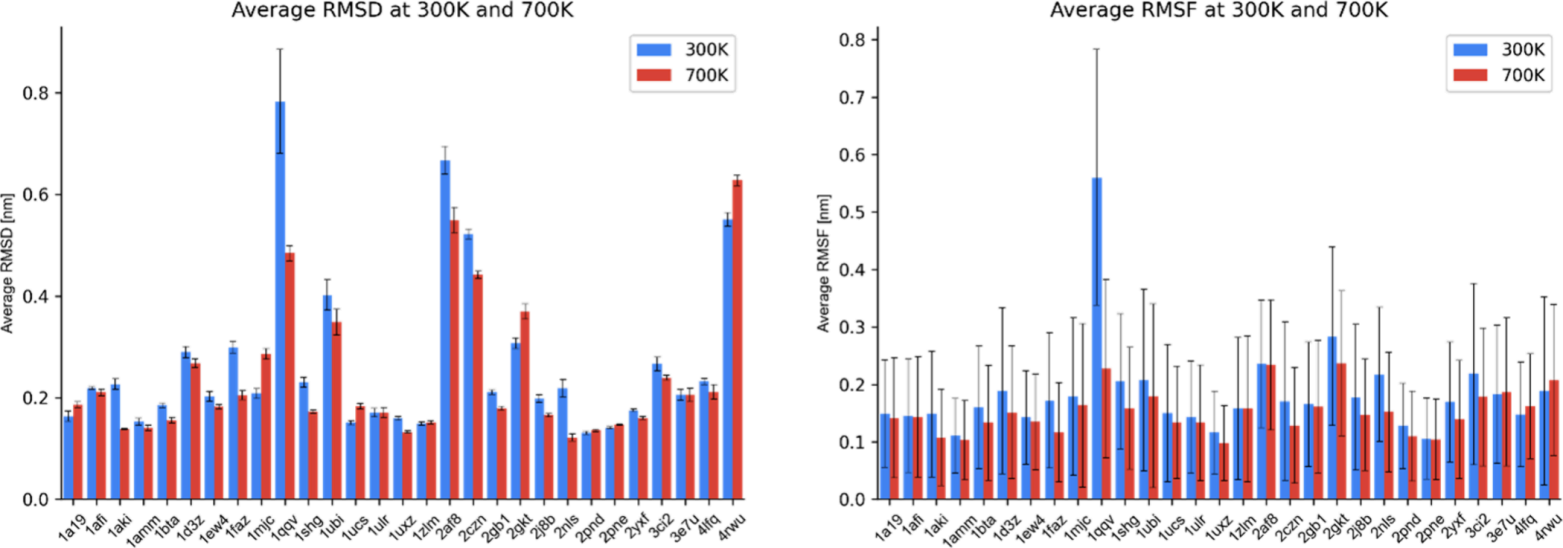
Average RMSD (left) and average RMSF (right) of each protein at 300 and 700 K over the entire time span of the MD trajectory (100 ns).

To investigate the effects of MW heating on the characteristics of proteins, we analyzed and compared the MD trajectories under equilibrium 300 K and nonequilibrium 700 K conditions (Figure 1). Specifically, we calculated structural properties, including the root-mean-square deviations (RMSD) with respect to reference protein structures obtained from experimental X-ray crystallography data, the root-mean-square fluctuations around the average atomic positions (RMSF), the intra- and intermolecular hydrogen bond statistics, the head-to-tail distances, and the radii of gyration of each protein. Moreover, we quantified van der Waals and electrostatic interactions within the protein, as well as between the protein and solvent. A summary of these results is displayed in Tables 1 and 2, while per-protein data is depicted in Supporting Information in Chart S1 through Chart S14. Statistical uncertainties on the per-protein data were obtained from a block averaging approach,^50^ as implemented in GROMOS++.^51^ For each protein, the decrease/increase was also determined as the average value at 300 K divided by the corresponding average value at 700 K, subsequently subtracted by 1. The standard error of the mean for the decrease/increase was calculated as the standard deviation of the per-protein ratio divided by the square root of the number of measurements per protein.

**Table 1:** Average Values of the Structural Analyses of the 29 Investigated Proteins

**analysis**	300 K	700 K	**decrease/increase**
RMSD (nm)^a^	0.270 ± 0.022	0.240 ± 0.010	–13.1% ± 4.5%
RMSF (nm)^b^	0.184 ± 0.122	0.153 ± 0.109	–19.0% ± 5.2%
RGYR (nm)^c^	1.237 ± 0.003	1.212 ± 0.003	–2.0% ± 0.3%
H2T (nm)^d^	2.002 ± 0.071	1.893 ± 0.064	–10.0% ± 4.6%
*n*_HB_^PPbb^^e^	56.8 ± 0.7	57.5 ± 0.7	1.6% ± 1.3%
*n*_HB_^PP^^f^	388.2 ± 0.9	393.5 ± 1	1.2% ± 0.2%
*n*_HB_^PW^^g^	396.7 ± 2.2	329.1 ± 1.8	–20.7% ± 0.8%
SASA (nm^2^)^h^	38.991 ± 0.214	35.297 ± 0.278	–10.5% ± 0.5%
SASA (polar)(nm^2^)^i^	30.028 ± 0.194	26.832 ± 0.228	–12.1% ± 0.6%
SASA (nonpolar)(nm^2^)^j^	9.015 ± 0.115	8.577 ± 0.127	–5.1% ± 1.3%

a Average MD trajectory protein backbone RMSD value compared to the experimentally determined protein structure.

b Average MD trajectory RMSF of the protein backbone.

c Average radius of gyration of proteins.

d Average head-to-tail distance of proteins.

e Average number of structure-forming intraprotein hydrogen bonds.

f Average number of total intraprotein hydrogen bonds.

g Average number of intermolecular hydrogen bonds between the protein and water molecules.

i Solvent-accessible surface area of all protein amino acid residues.

h Solvent-accessible surface area of polar protein amino acid residues.

j Solvent-accessible surface area of nonpolar protein amino acid residues.

**Table 2:** Average Intraprotein and Protein–Water Interaction energies (in kJ/mol)

	300 K	**700 K**	**decrease/increase**
*E*_vdw_^PP^^a^	–2125 ± 4	–2101 ± 4	1.3% ± 0.2%
*E*_es_^PP^^b^	–6594 ± 31	–7248 ± 37	–9.3% ± 0.7%
*E*_tot_^PP^^c^	–8719 ± 31	–9349 ± 35	–7.0% ± 0.6%
*E*_vdw_^PW^^d^	–352 ± 4	–568 ± 5	–39.1% ± 2.2%
*E*_es_^PW^^e^	–11,142 ± 59	–8250 ± 54	36.5% ± 1.3%
*E*_tot_^PW^^f^	–11,493 ± 57	–8818 ± 52	31.4% ± 1.1%

a Intramolecular intraprotein interaction energy—van der Waals contribution.

b Intramolecular intraprotein interaction energy—electrostatic contribution.

c Intramolecular intraprotein interaction energy.

d Intermolecular protein–water interaction energy—van der Waals contribution.

e Intermolecular protein–water interaction energy—electrostatic contribution.

f Intermolecular protein–water interaction energy.

Given that comparing the RMSD values of structures with a different number of amino acid residues can be misleading due to the inherent size dependence of positional RMSD, we normalized our results to correct for the number of amino-acid residues in each protein structure. Specifically, we employed the formula derived by Carugo and Pongor^52^ that translates the RMSD value of any protein structure into the equivalent RMSD value for a 100-residue protein structure, which we term RMSD_100_. The equation we used is as follows:
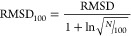
1where RMSD represents the original RMSD value and *N* is the number of amino-acid residues in the protein structure. This approach ensures a meaningful comparison between structures of different sizes, with the caveat that this method has not been validated for protein structures with less than 40 amino acid residues.

During MW radiation, rotational excitation of polar solvent molecules induces changes in the structural properties of proteins. In our MW MD simulations, the proteins’ temperature was maintained at 300 K, even as the rotational temperature of the solvent was increased to 700 K. This fact underscores that the observed structural modifications are likely attributable to direct interactions between proteins and solvent molecules. Consistent with the β-peptide analysis,^13^ our data reveal an average destabilization of 20.7% in the number of hydrogen bonds interfacing the proteins with the solvent. Concurrently, the level of protein hydration decreases at the higher rotational temperature of 700 K, augmenting the strength of intraprotein hydrogen bonds. This is attested by a slight but significant rise of 1.6% in the average number of structure-forming intraprotein hydrogen bonds and a 1.2% overall increase in the number of intraprotein hydrogen bonds. These results thus suggest that the proteins’ intramolecular hydrogen bond network becomes more robust in the context of the MW-elevated rotational temperatures.

**Figure 2:**
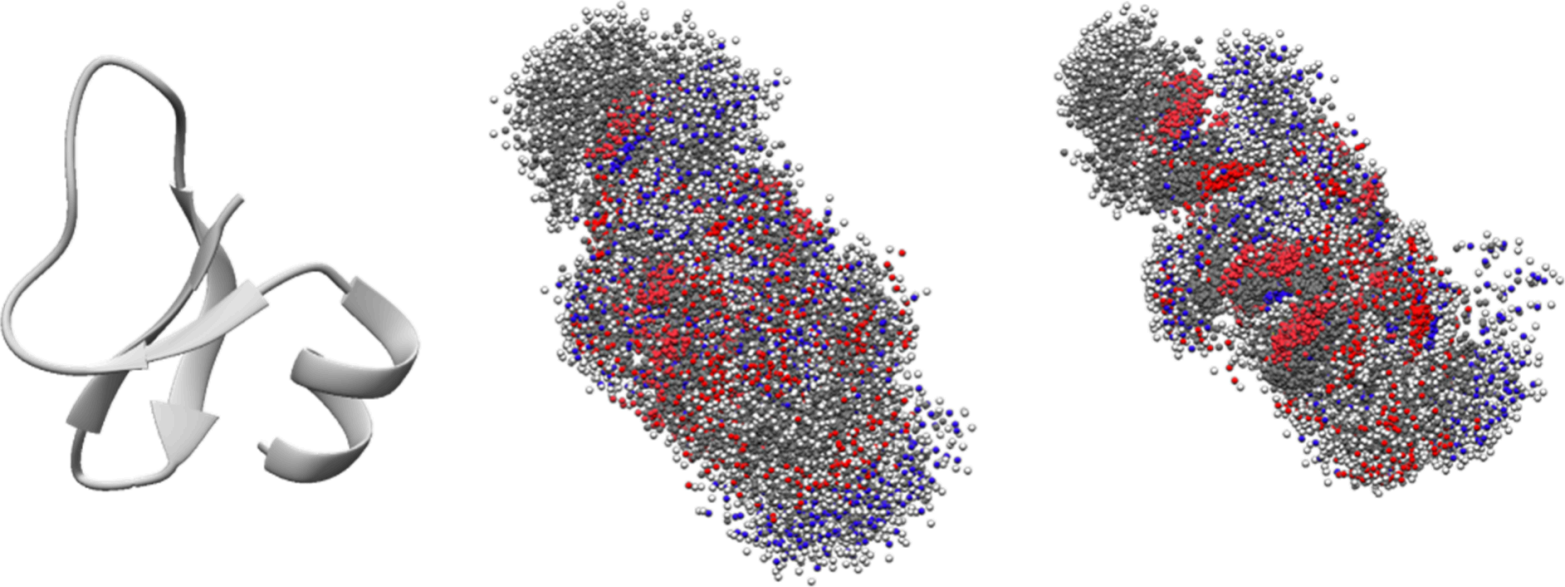
Superpositions of protein (PDB ID: 2NLS) atoms over the entire time span of the MD trajectory (100 ns). Displayed are the experimental structure in cartoon (left) and the structures for equilibrium MD simulations at 300 K (center) and for MW heating with a nonequilibrium rotational temperature of 700 K (right).

The enhanced stability of the intramolecular hydrogen bond network likely causes proteins to adopt more compact conformations, as depicted in Figure 2, which presents the superposition of protein atoms over the course of the MD trajectories. As evident from the figure, the protein structure appears denser when it is subjected to MW heating. Specifically, at the elevated rotational temperature of 700 K, the average RMSD value decreases to 0.240 nm, translating to a 13.1% reduction from the RMSD at 300 K. Similarly, the average RMSF of the protein backbone decreases to 0.153 nm at 700 K, representing a 19.0% reduction relative to the RMSF at 300 K. Furthermore, the average radius of gyration shrinks to 1.212 nm at 700 K, forming a modest 2.0% decrease from 300 K, which can be associated with the enhanced compactness of the protein structure at higher rotational temperatures. Moreover, the average head-to-tail distance shortens to 1.893 nm at 700 K, representing a significant decrease of 10.0% from 300 K. Collectively, these outcomes point to the substantial impact of MW heating on the structural properties of proteins.

Concomitantly, the solvent-accessible surface area decreases by 10.5% under MW conditions. Intriguingly, nonpolar amino acids (Ala, Cys, Ile, Leu, Met, Phe, Trp Tyr, and Val) experience a more pronounced reduction (12.1%) at higher rotational temperatures compared with their polar (Arg, Asn, Asp, Glu, Gly, Gln, His, Lys, Pro, Ser, and Thr) counterparts (5.1%). This difference likely arises because water tends to adopt a less polar character under MW conditions, as suggested by our data. This could lead polar amino acid residues to minimize their exposure to the increasingly apolar solvent environment, while the nonpolar amino acid residues remain comparatively less perturbed.

Conversely, the effects on the secondary structure elements under equilibrium and MW conditions were found to be less pronounced or even insignificant. The frequency of helices displayed a minor increase of 1.7% ± 5.1% under MW conditions, a statistically insignificant shift. The frequency of beta sheets was observed to reduce by 3.8% ± 3.5%, and the incidence of undefined structures decreased by 1.2% ± 1.3%. These relatively minor alterations in the secondary structures suggest that the major structural changes detected at higher rotational temperatures largely involve shifts in the proteins’ tertiary structure. To get a complete picture of the MW irradiation effects on the structure of proteins, one must also consider the alterations in intraprotein and protein–solvent interactions, presented in Table 2.

Under MW heating, the total intramolecular nonbonded energy of the proteins decreased by 7.0%, on average, indicating strengthened interactions within proteins. The largest contribution to this change is due to the strengthened electrostatic interactions (9.3%), while the van der Waals interactions weakened by 1.3%. The stronger intramolecular interactions under MW conditions are a consequence of more intraprotein hydrogen bonds (1.2%) and the shorter average distance of these hydrogen bonds (by 1.0%; data not shown). This trend coincides with a previous β-peptide study,^13^ which shows a 7.3% decrease in intramolecular interaction energies under MW conditions, which originated almost exclusively from electrostatic interactions through the reinforcement of intramolecular hydrogen bonds.

Concomitantly, the interactions between the proteins and solvent were weakened by 31.4%, on average, under MW radiation. The largest contribution to this change is due to the weakened electrostatic interactions (36.5%) as a result of the solvent becoming less polarized due to faster rotations of water molecules. In contrast, since their contribution is minimal, the van der Waals interactions between the proteins and solvent strengthened by 39.1% but had a minimal effect on the total protein–solvent interactions. The previous β-peptide study showed no change in peptide-solvent van der Waals contributions, arguably because the β-peptide was much shorter and less structured than the proteins in our study. In a structured protein, less polar MW-heated water molecules can occupy smaller cavities on the protein surface and interact favorably in terms of the van der Waals interactions.

## 4 Conclusions

The present study utilized MD simulations to investigate the impact of MW irradiation on the protein structure and energetics. By increasing the rotational temperature to 700 K while maintaining the protein temperature at 300 K, we aimed to delineate structural and energetic changes induced specifically by MW heating rather than bulk temperature effects.

The results reveal that MW irradiation in the 300 MHz to 20 GHz frequency range prompts notable modifications in the tertiary structure of proteins. Remarkably, these changes occur with minimal impact on the secondary structure elements, such as unfolding,^27^ within a 100 ns time frame. Under MW conditions, proteins tend to adopt more compact conformations, as evidenced by decreased RMSD, RMSF, head-to-tail distance, and radius of gyration. The observed compaction appears to arise from the strengthening of intramolecular interactions, particularly electrostatic interactions and hydrogen bonds within the protein. Concurrently, the protein–solvent hydrogen bond network becomes destabilized under MW-induced heating, presumably causing contraction of the protein structure.

The reinforcement of intramolecular hydrogen bonds and electrostatic interactions indicates that the protein structure becomes more robust under MW irradiation. As the water takes on less polar character at elevated rotational temperatures, polar amino acid residues decrease solvent exposure by moving to the protein interior. The resulting dense, tightly packed conformations enhance the structural integrity of the protein in the altered solvation environment.

However, the limitations of our replacement model for MW radiation should be acknowledged. This model does not account for the direct effect of MW on proteins, which may introduce artifacts. Previous MD simulation studies incorporating oscillating electric fields^53,54^ have demonstrated disruption in hydrogen bonding, especially for charged residues, which undergo more localized motion. This suggests that protein groups such as −OH, −NH_2_, and −COOH could be affected by MW; therefore, it is conceivable that if charged protein groups were exposed to a sufficiently strong external electromagnetic field, more hydrogen bond breaking could lead to more substantial and irreversible changes in protein structure changes.

While this study primarily focused on the influence of MW heating on protein structure, the downstream functional implications of these structural changes remain to be fully elucidated. Compaction and rigidification of the protein structure might impede the dynamics and conformational transitions necessary for protein function. Additionally, the reduced solvent accessibility of polar amino acid residues under MW conditions could potentially influence interprotein interactions, ligand binding, and enzymatic activity.

## Data Availability

The complete topology and input files for the proteins are available at https://github.com/maticbroz/effect-of-microwaves-on-protein-structure.

## Supporting Information Available

Supporting Information.docx The Supporting Information is available free of charge at https://pubs.acs.org/doi/10.1021/acs.jcim.3c01937.

Table S1: List of all 29 X-ray and NMR protein structures used in this study; Chart S1: Average radius of gyration of each protein at 300 and 700 K; Chart S2: Average head-to-tail distance of each protein at 300 and 700 K; Chart S3: Average number of backbone hydrogen bonds of each protein at 300 and 700 K; Chart S4: Average number of intraprotein hydrogen bonds of each protein at 300 and 700 K; Chart S5: Average number of hydrogen bonds between protein and water of each protein at 300 and 700 K; Chart S6: Average solvent-accessible surface area of each protein at 300 and 700 K; Chart S7: Average solvent-accessible surface area of polar amino acid residues of each protein at 300 and 700 K; Chart S8: Average solvent-accessible surface area of nonpolar amino acid residues of each protein at 300 and 700 K; Chart S9: Average intraprotein van der Waals interaction energy of each protein at 300 and 700 K; Chart S10: Average intraprotein electrostatic interaction energy of each protein at 300 and 700 K; Chart S11: Average intraprotein interaction energy of each protein at 300 and 700 K; Chart S12: Average protein–water van der Waals interaction energy of each protein at 30 and 700 K; Chart S13: Average protein–water electrostatic interaction energy of each protein at 300 and 700 K; Chart S14: Average protein–water interaction energy of each protein at 300 and 700 K (PDF)

## ABBREVIATIONS

MW: Microwave
MD: Molecular dynamics
RMSD: Root-mean-square deviation
RMSF: Root mean square fluctuation
RGYR: Radius of gyration
H2T: Head-to-tail distance
*n*_HB_^PBB^: Number of backbone intraprotein hydrogen bonds
*n*_HB_^PP^: Number of total intraprotein hydrogen bonds
*n*_HB_^PW^: Number of intermolecular hydrogen bonds between protein and water
SASA: Solvent accessible surface area
*E*_vdw_^PP^: Intraprotein van der Waals interaction energy
*E*_es_^PP^: Intraprotein electrostatic interaction energy
*E*_tot_^PP^: Total intraprotein interaction energy
*E*_vdw_^PW^: Protein–water van der Waals interaction energy
*E*_es_^PW^: Protein–water electrostatic interaction energy
*E*_tot_^PW^: Total protein–water interaction energy
