# Supplementary material for: The Effect of Microwaves on Protein Structure: Molecular Dynamics Approach
Source: J Chem Inf Model. 2024 Mar 13;64(6):2077–83. doi: 10.1021/acs.jcim.3c01937 (PMC10966651; doi:10.1021/acs.jcim.3c01937)
Supplement: Supplementary file 1 — ci3c01937_si_001.pdf [file ci3c01937_si_001.pdf]

The Effect of Microwaves on Protein Structure: Molecular Dynamics Approach

Matic Broz[1], *Chris Oostenbrink*[*,2], *Urban Bren*[1,3,4,*]

[1] Faculty of Chemistry and Chemical Engineering, University of Maribor, Smetanova ulica 17, SI-2000 Maribor, Slovenia;

[2] Institute of Molecular Modeling and Simulation, University of Natural Resources and Life Sciences, Muthgasse 18, 1190 Vienna, Austria;

[3] Faculty of Mathematics, Natural Sciences and Information Technologies, University of Primorska, Glagoljaška ulica 8, SI-6000 Koper, Slovenia;

[4] Institute of Environmental Protection and Sensors, Beloruska ulica 7, SI-2000 Maribor, Slovenia;

* To whom the correspondence should be addressed: chris.oostenbrink@boku.ac.at and urban.bren@um.si

Table S1: List of all 29 X-ray and NMR protein structures used in this study. **NR**: Number of residues. *****: Has a paired NMR structure.

PDB ID	Protein name	Organism	NR	Method
1A19*	Barstar, mutant C82A	Bacillus amyloliquefaciens	90	X-ray
1AFI	Mercury binding protein (MerP)	Shigella flexneri	72	NMR
1AKI*	Lysozyme	Gallus gallus	129	X-ray
1AMM	γ B Crystallin	Bos taurus	174	X-ray
1BTA	Barstar	Bacillus amyloliquefaciens	89	NMR
1D3Z	Ubiquitin	Homo sapiens	76	NMR
1EW4	CyaY	Escherichia coli	106	X-ray
1FAZ	Phospholipase A2	Streptomyces	122	X-ray
1MJC	Major cold shock protein	Escherichia coli	69	X-ray
1QQV	Villin headpiece	Gallus gallus	67	NMR
1SHG	α-spectrin, SH3-domain	Gallus gallus	62	X-ray

1UBI	Ubiquitin	Homo sapiens	76	X-ray
1UCS	Antifreeze peptide RD1	Lycodichthys dearborni	64	X-ray
1ULR	Putative acylphosphatase	Thermus thermophilus	88	X-ray
1UXZ	Cellulase B	Cellvibrio mixtus	131	X-ray
1ZLM	Osteoclast stimulating factor 1, SH3-domain	Homo sapiens	58	X-ray
2AF8	Actinorhodin polyketide synthase acyl carrier protein	Streptomyces coelicolor	86	NMR
2CZN	Chitinase	Pyrococcus furiosus	103	NMR
2GB1	Protein G, B1 domain	Streptococcus sp. ‘group g’	56	NMR
2GKT	Turkey ovomucoid third domain	Meleagris gallopavo	51	X-ray
2J8B	Human CD59 glycoprotein	Homo sapiens	79	X-ray
2NLS	Human β-defensin-1, mutant Q24A	Homo sapiens	36	X-ray
2PND	Murine CRIg	Mus musculus	119	X-ray
2PNE	Glycine-rich antifreeze protein	Hypogastrura harveyi	81	X-ray
2YXF	β-2-microglobulin	Homo sapiens	100	X-ray
3CI2	Chymotrypsin inhibitor 2 (CI-2)	Hordeum vulgare	66	NMR
3E7U	Plectasin	Pseudoplectania nigrella	40	X-ray

4LFQ	Potassium channel toxin L-ShK	Stichodactyla helianthus	35	X-ray
4RWU	Protein Sis1	Saccharomyces cerevisiae s288c	92	X-ray

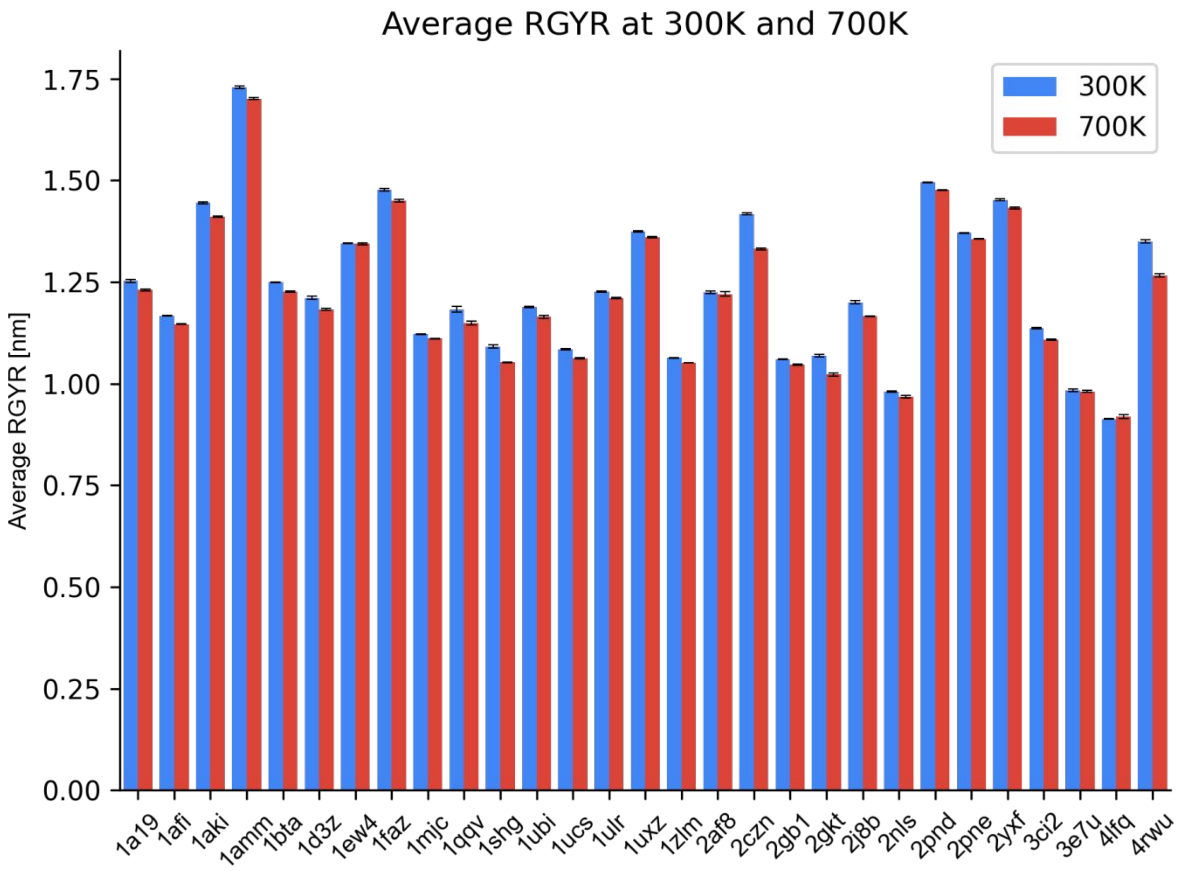

Chart S1: Average radius of gyration of each protein at 300 and 700 K.

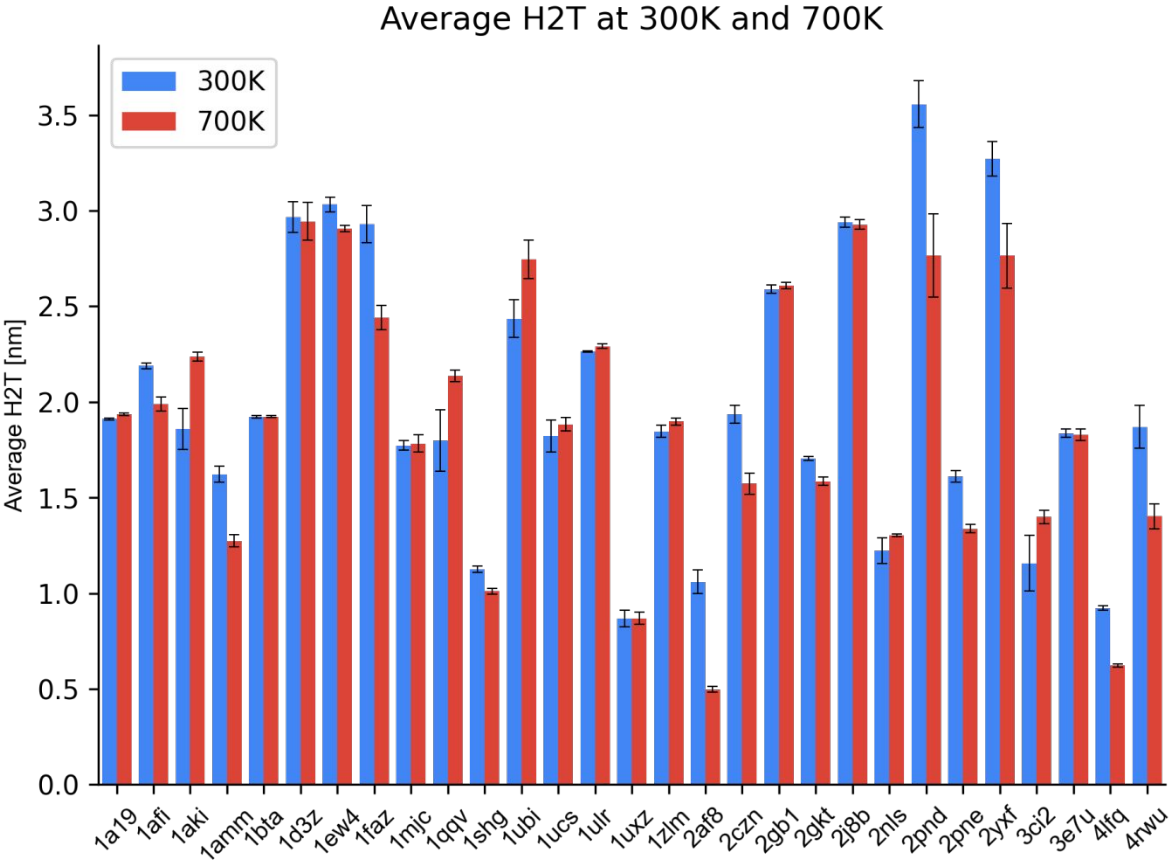

Chart S2: Average head-to-tail distance of each protein at 300 and 700 K.

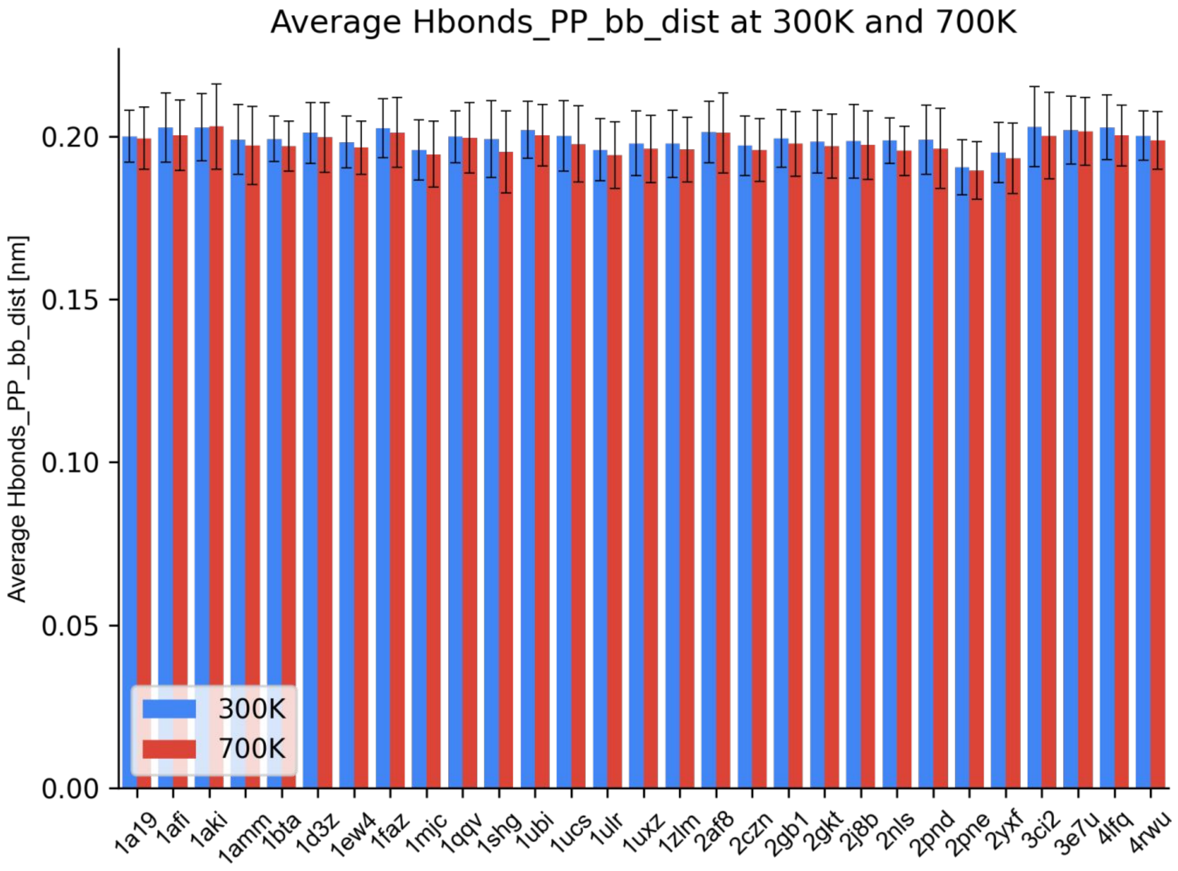

Chart S3: Average number of backbone hydrogen bonds of each protein at 300 and 700 K.

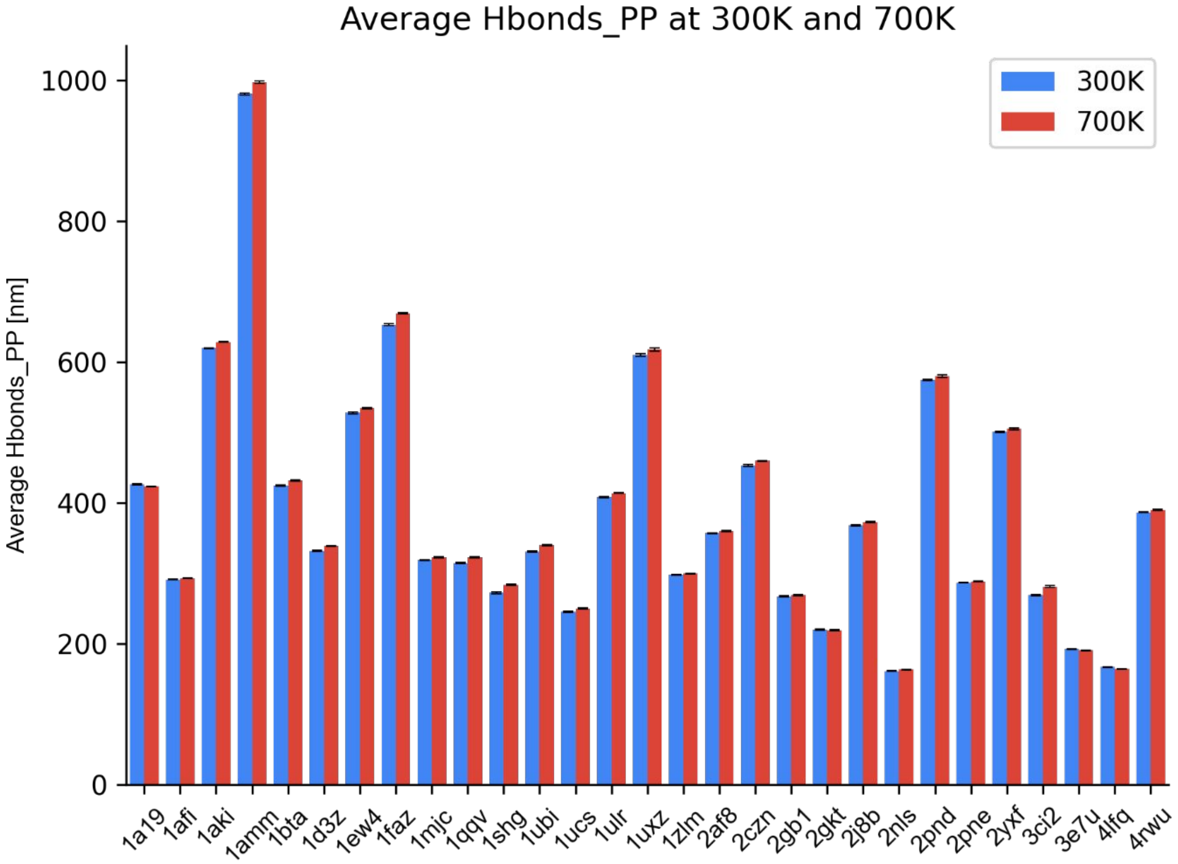

Chart S4: Average number of intraprotein hydrogen bonds of each protein at 300 and 700 K.

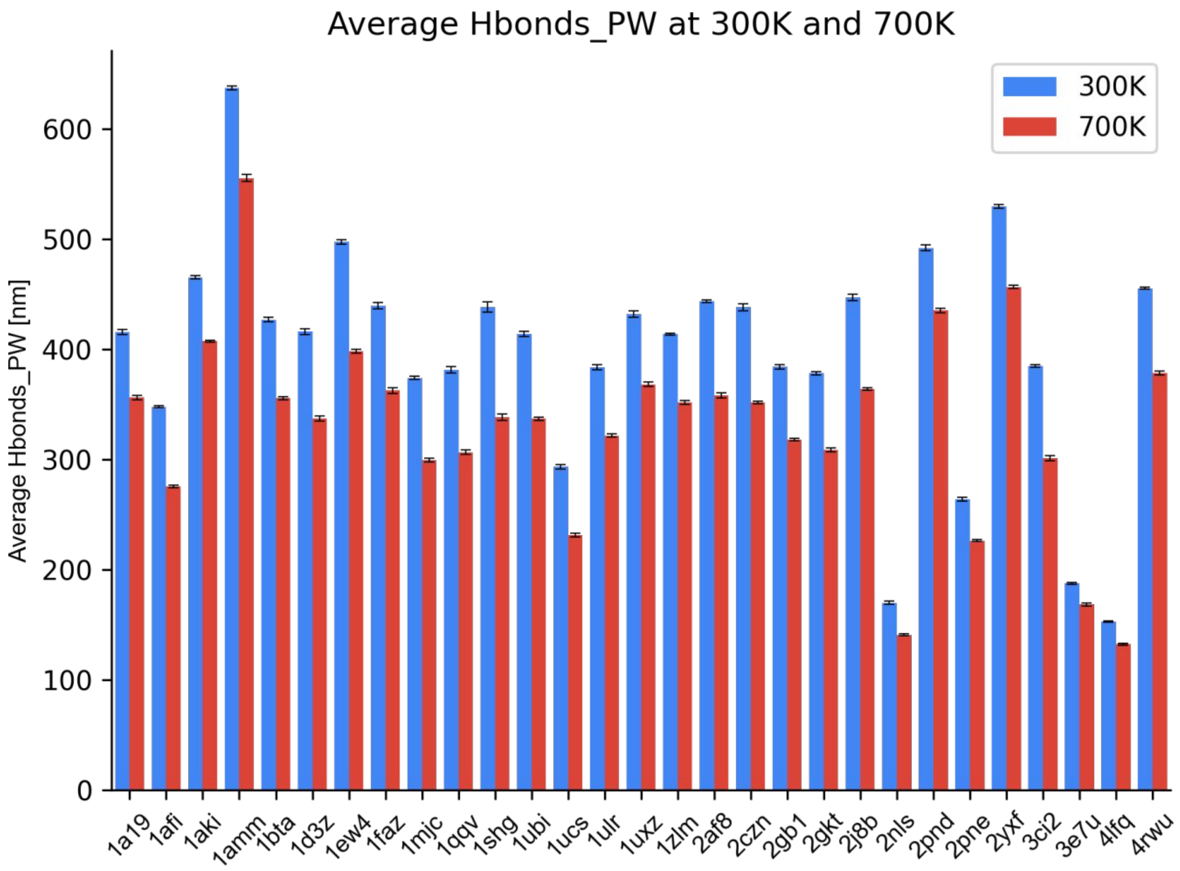

Chart S5: Average number of hydrogen bonds between protein and water of each protein at 300 and 700 K.

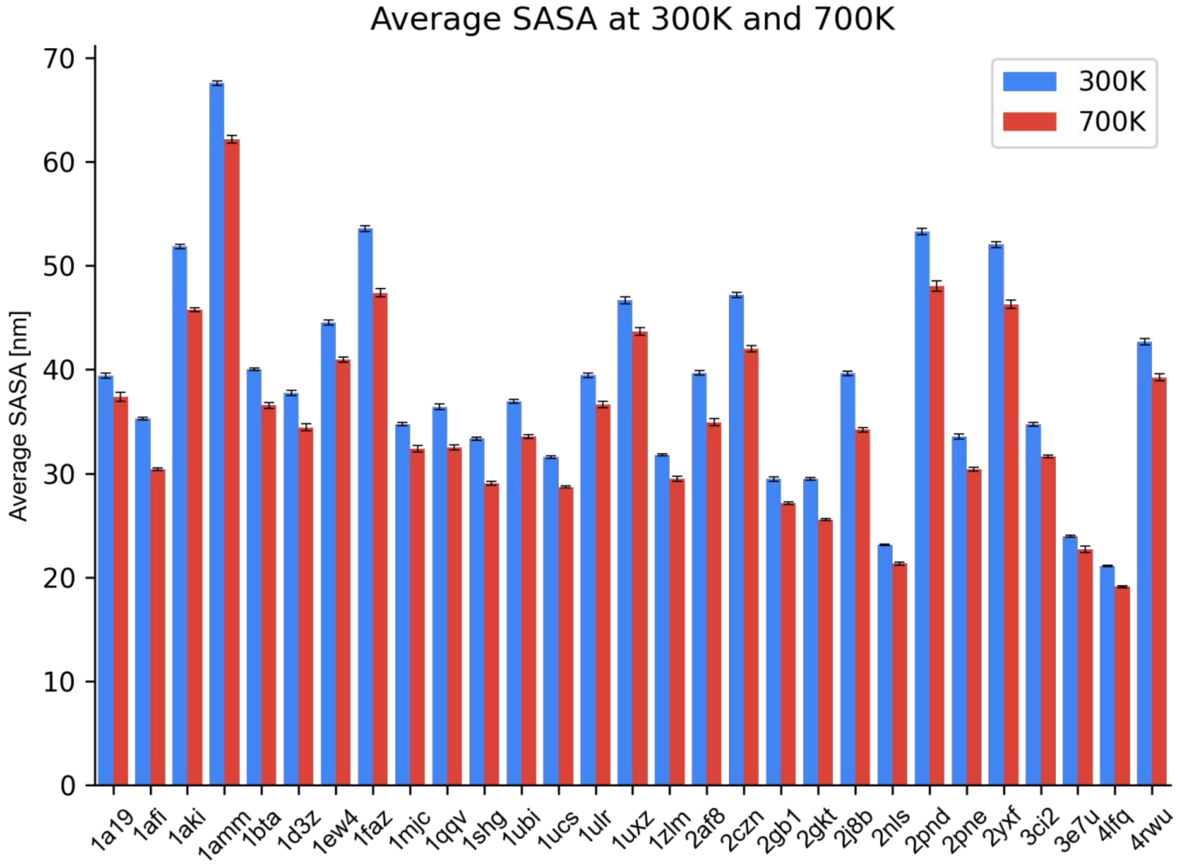

Chart S6: Average solvent-accessible surface area of each protein at 300 and 700 K.

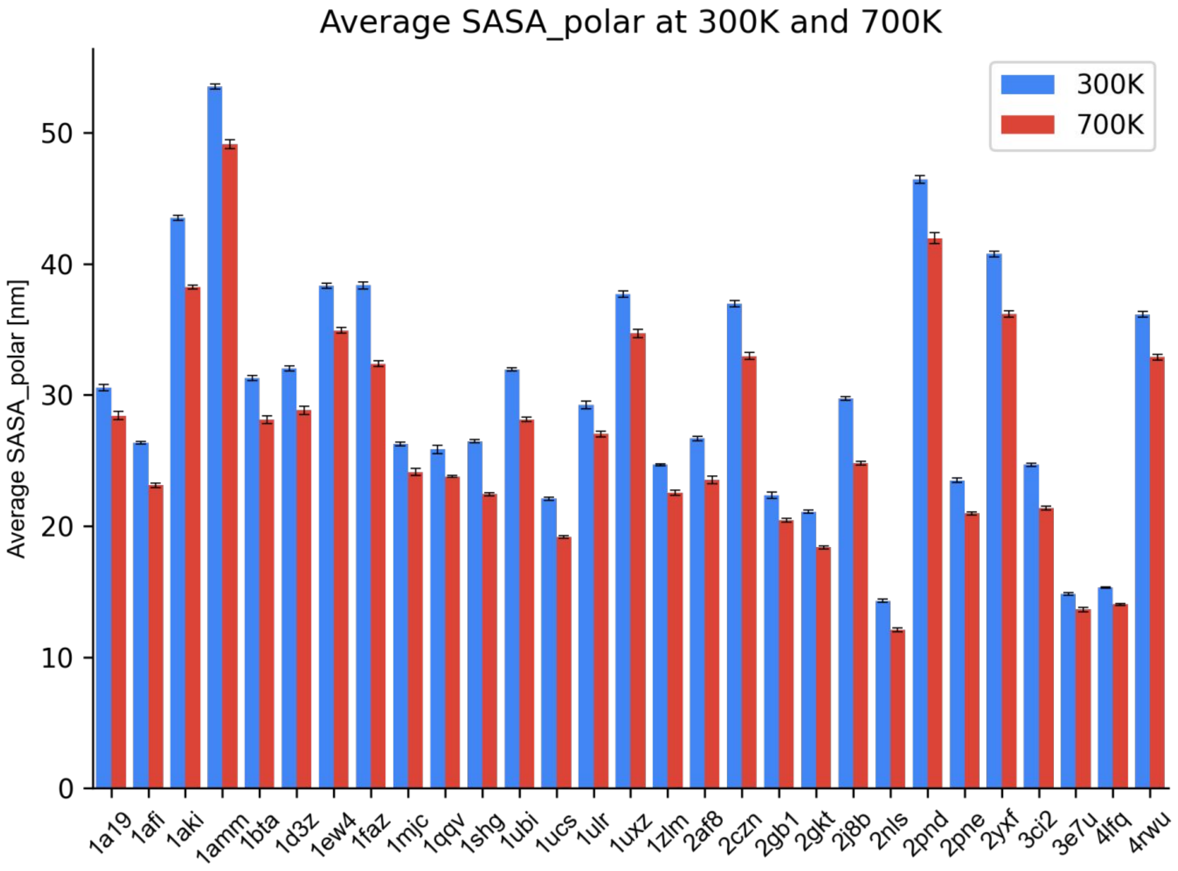

Chart S7: Average solvent-accessible surface area of polar amino acid residues of each protein at 300 and 700 K.

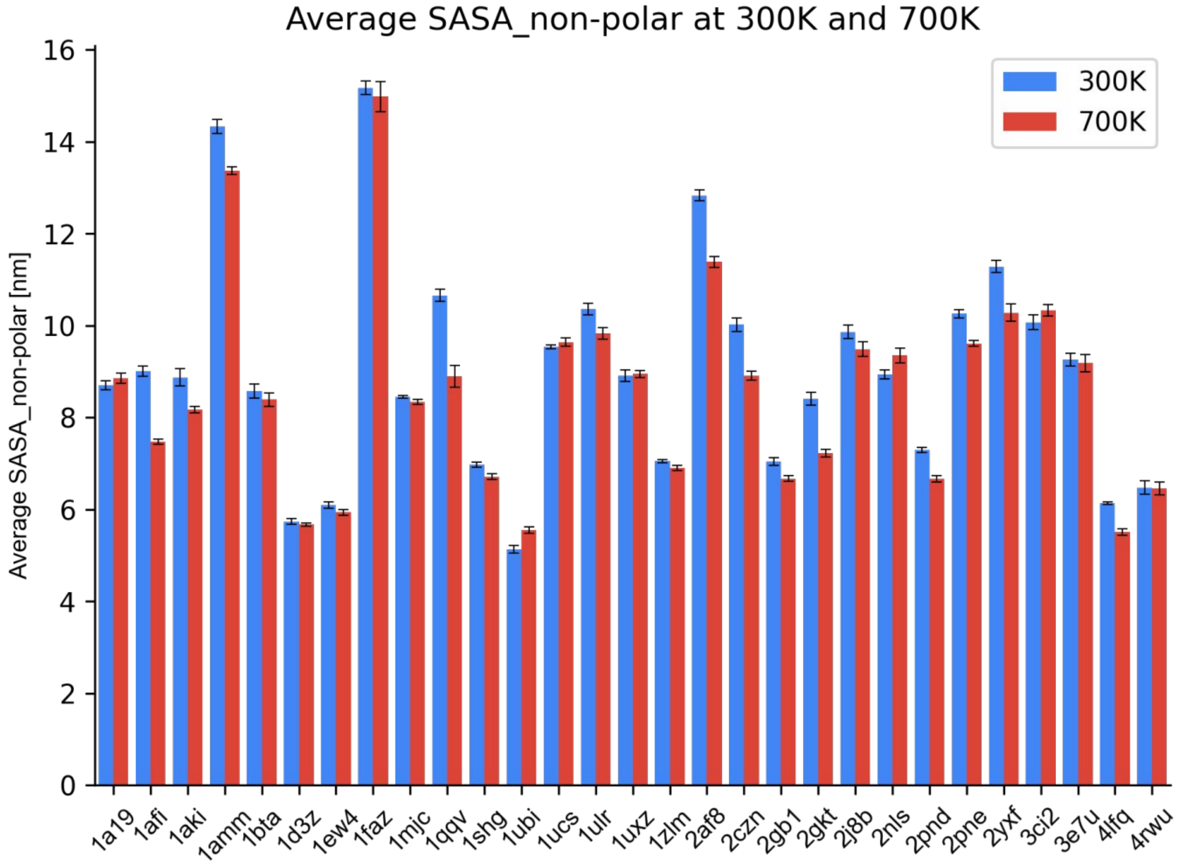

Chart S8: Average solvent-accessible surface area of nonpolar amino acid residues of each protein at 300 and 700 K.

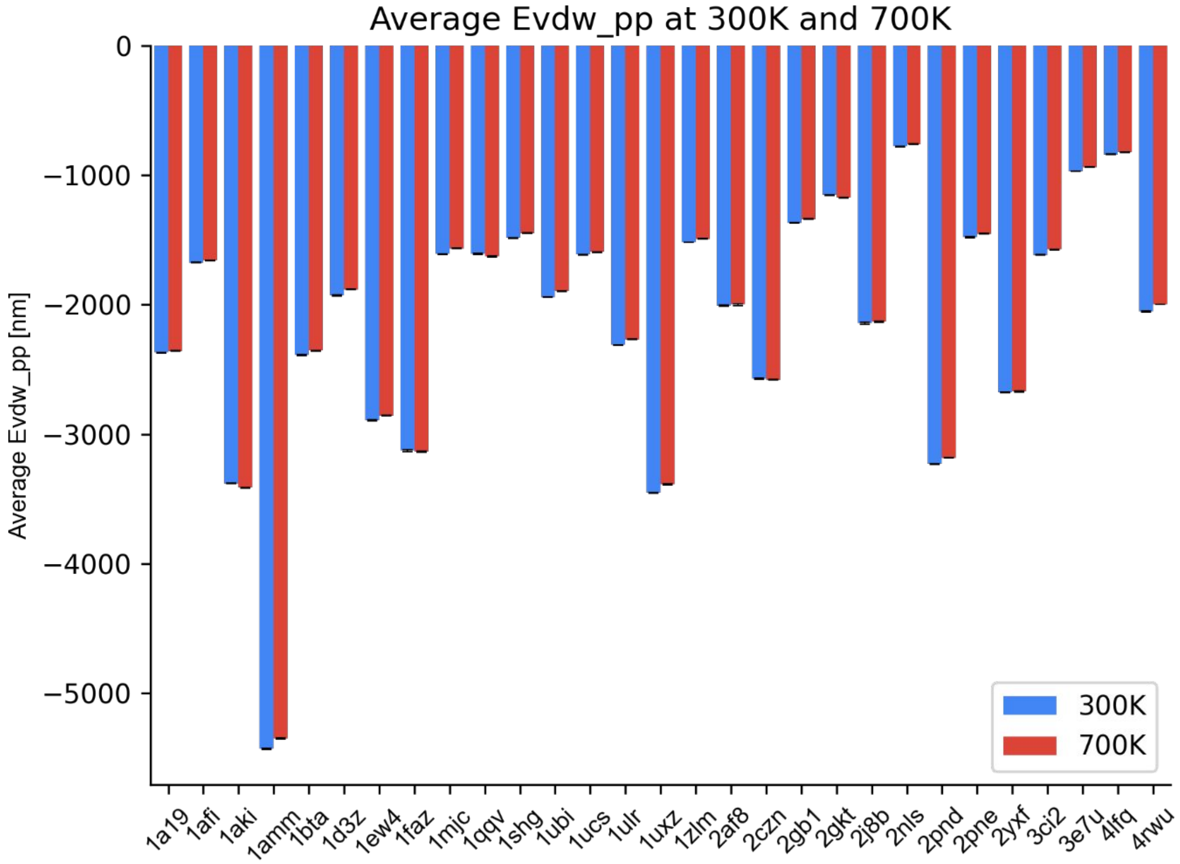

Chart S9: Average intraprotein van der Waals interaction energy of each protein at 300 and 700 K.

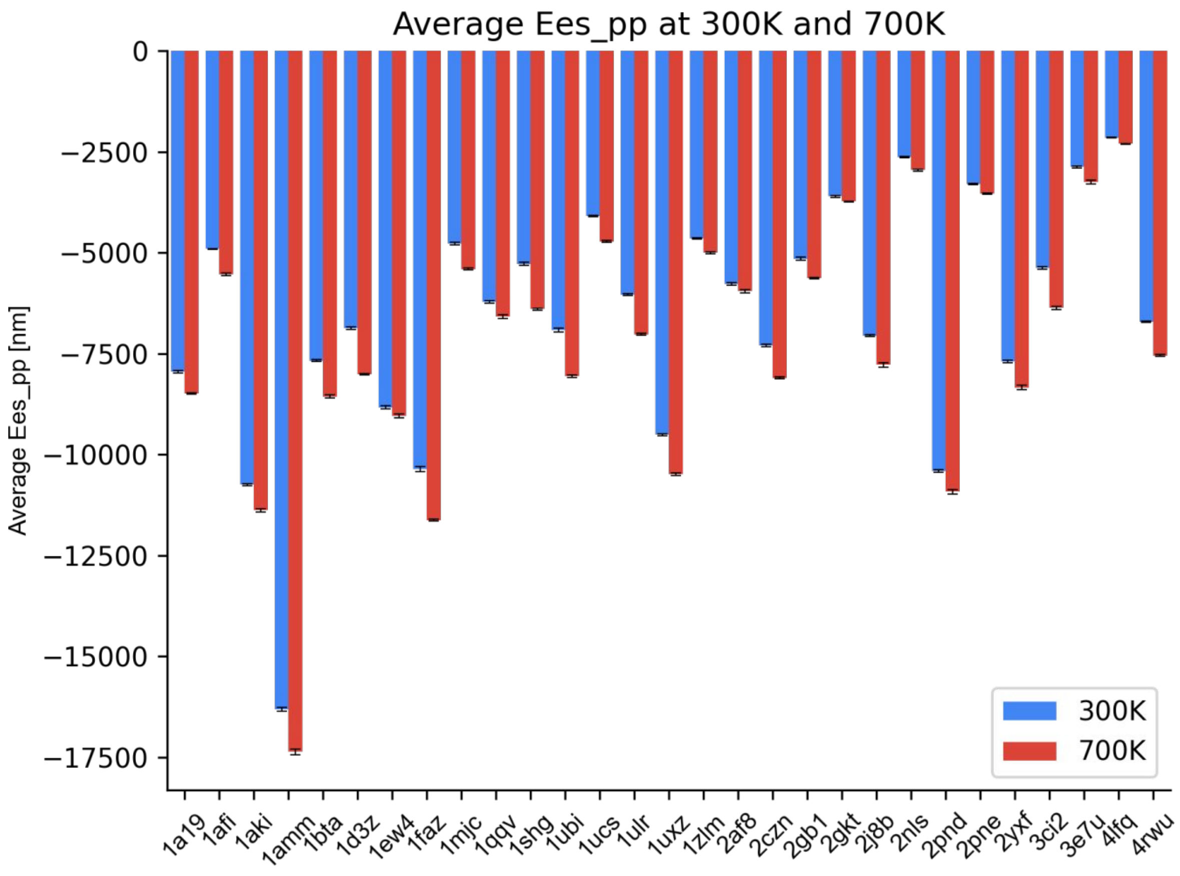

Chart S10: Average intraprotein electrostatic interaction energy of each protein at 300 and 700 K.

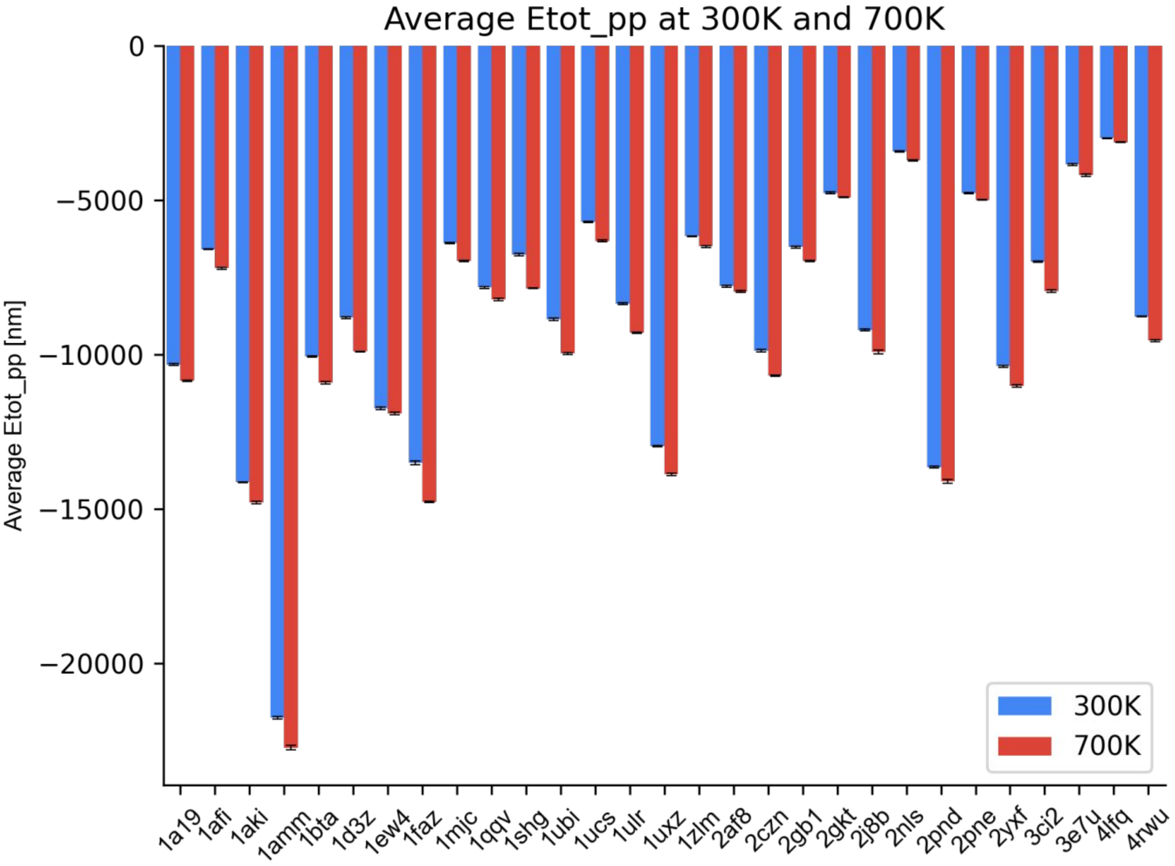

Chart S11: Average intraprotein interaction energy of each protein at 300 and 700 K.

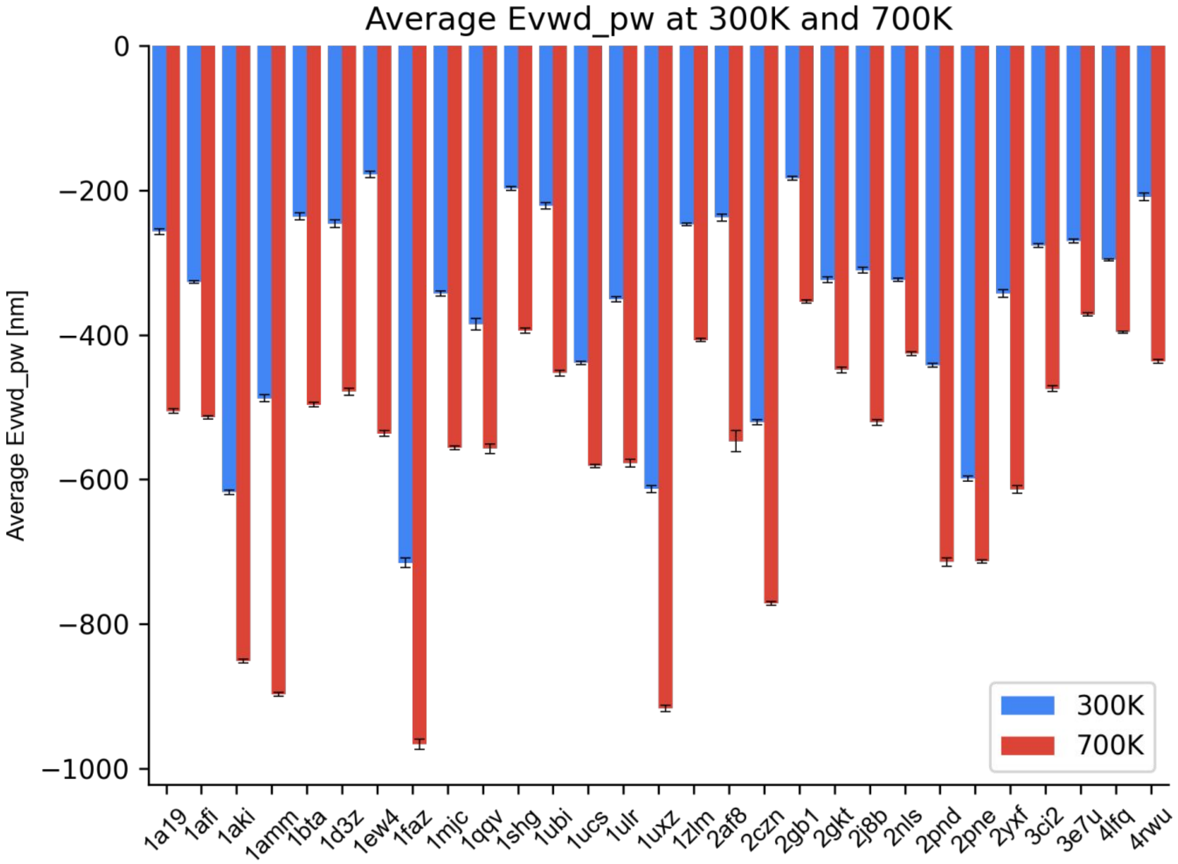

Chart S12: Average protein-water van der Waals interaction energy of each protein at 300 and 700 K.

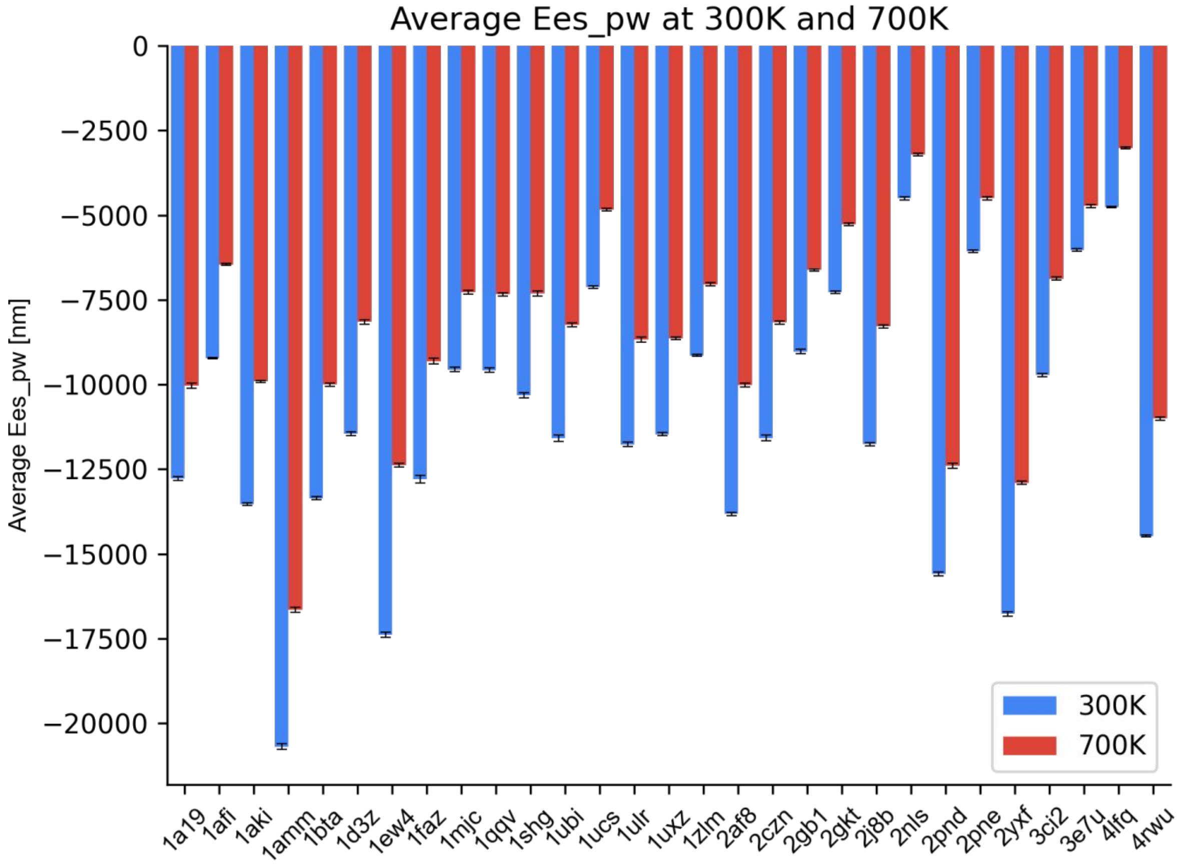

Chart S13: Average protein-water electrostatic interaction energy of each protein at 300 and 700 K.

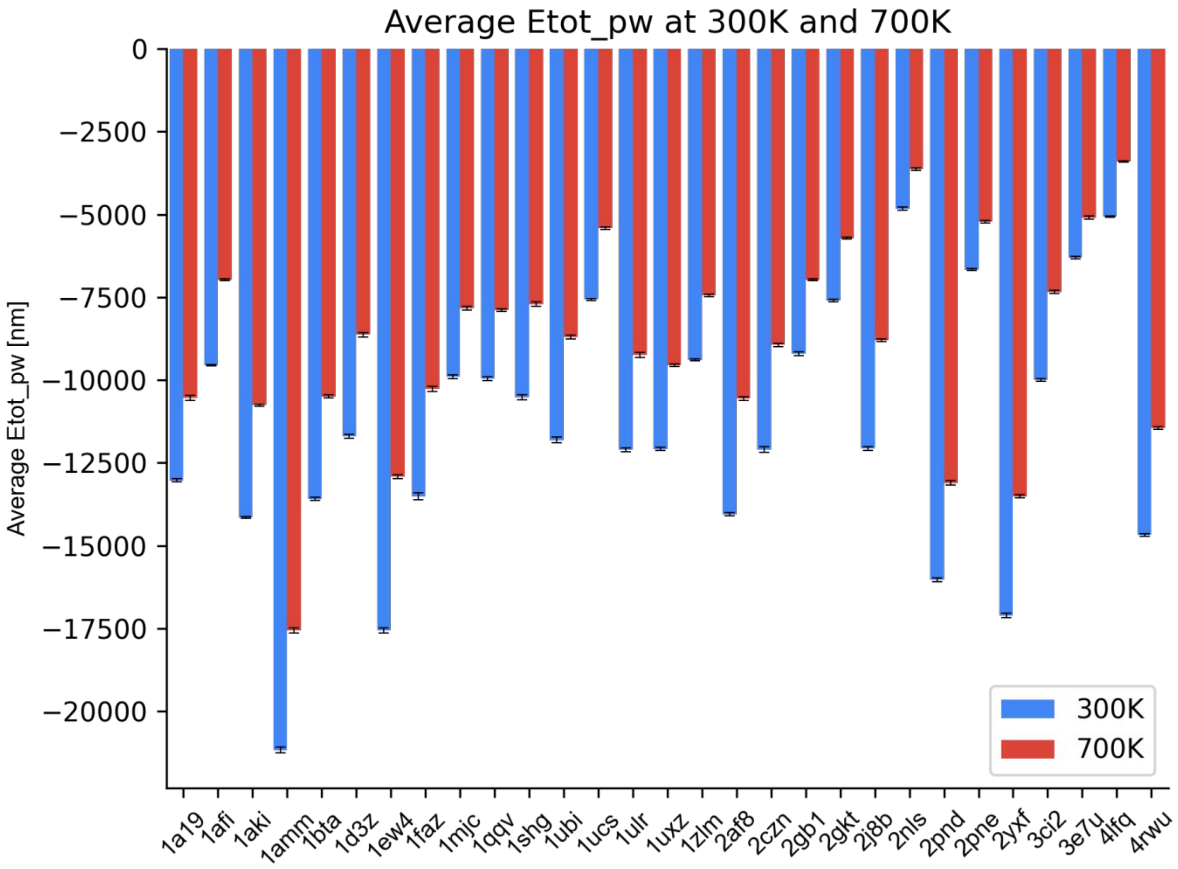

Chart S14: Average protein-water interaction energy of each protein at 300 and 700 K.
